# Phytochemical Characterization of *Hibiscus tiliaceus* L. Leaves and Evaluation of Their Antisickling, Antioxidant, and Anti-Inflammatory Activities

**DOI:** 10.3390/molecules30081765

**Published:** 2025-04-15

**Authors:** Marguerite Borive Amani, Michel Frederich, Olivia Jansen, Olivier Bonnet, Allison Ledoux, Patrick B. Memvanga, Salomon Batina Agasa, Ange Mouithys-Mickalad, Roland Marini Djang’eing’a

**Affiliations:** 1 Department of Pharmacy, Faculty of Medicine and Pharmacy, University of Kisangani, Kisangani 2012, Democratic Republic of the Congo; maguyborive@gmail.com; 2 Department of Pharmacy, Laboratory of Pharmacognosy, Center for Interdisciplinary Research on Medicines (CIRM), University of Liège (ULiège), 4000 Liège, Belgium; m.frederich@uliege.be (M.F.); olivier.bonnet@uliege.be (O.B.); allison.ledoux@uliege.be (A.L.); 3 Department of Pharmacy, Laboratory of Pharmaceutical Analytical Chemistry, Center for Interdisciplinary Research on Medicines (CIRM), University of Liège (ULiège), 4000 Liège, Belgium; 4 Centre de Recherche et d’Innovation Technologique en Environnement et en Sciences de la Santé (CRITESS), Faculty of Pharmaceutical Sciences, University of Kinshasa, Kinshasa XI 212, Democratic Republic of the Congo; patrick.memvanga@unikin.ac.cd; 5 Department of Internal Medicine, Faculty of Medicine and Pharmacy, University of Kisangani, Kisangani 2012, Democratic Republic of the Congo; agasasalomon@gmail.com; 6 Center for Oxygen Research and Development (CORD), University of Liège (Uliège), 4000 Liège, Belgium; amouithys@uliege.be

**Keywords:** phytochemical, sickle cell disease, *Hibiscus tiliaceus* L., aqueous extract, biological activities

## Abstract

Sickle cell disease (SCD) is a neglected tropical disease (NTD) associated with severe health consequences, including death. *Hibiscus tiliaceus* L., from the Malvaceae family, is used traditionally in Kisangani, Democratic Republic of the Congo (DRC), to alleviate symptoms of SCD. However, the specific phytochemicals responsible for the observed therapeutic effects remain unclear. This study aims to characterize the aqueous leaf extract of *H. tiliaceus* and assess its biological activity against sickle cell disease, including its antisickling, antioxidant, and anti-inflammatory effects. Using techniques such as TLC, HPLC-UV/DAD, LC-MS, and NMR, we identified kaempferol 3-*O*-rutinoside and rutin in the aqueous extract of *H. tiliaceus* leaves. Rutin exhibited potent antioxidant and anti-inflammatory activities, with IC_50_ values of 5 µg/mL and 2.5 µg/mL, respectively. Conversely, kaempferol 3-*O*-rutinoside demonstrated superior antisickling activity, normalizing sickled red blood cells with an IC_50_ < 12.5 µg/mL. Due to the pathophysiology of SCD, which involves the polymerization of red blood cells, which induces oxidative stress and an inflammatory response, this study suggests the importance of *H. tiliaceus* for the management of SCD. Additionally, the combined effect of molecules in *H. tiliaceus* will help in normalizing erythrocytes, inhibiting free radicals generated by early hemolysis, thus contributing to inflammatory processes reduction. This finding provides evidence and validates the traditional use of *H. tiliaceus* aqueous extract for the management of SCD.

## 1. Introduction

Neglected tropical diseases (NTDs) encompass a diverse range of conditions that have severe health and socio-economic impacts. They are associated with stigma and social exclusion due to hopeless conditions. NTDs have a high prevalence and mortality, particularly in poor communities in tropical areas, although some have a much wider geographic distribution [1].

According to the World Health Organization (WHO), NTDs can be classified into diseases of microbial infectious origin (viral, bacterial, fungal, etc.) and non-infectious origin. Sickle cell disease (SCD) is classified in the non-infectious class following the criteria of the Public Library of Science [1,2]. NTDs are characterized by (i) a global distribution with the highest burden among the poorest and most disadvantaged populations, (ii) a serious morbidity associated with reduced quality of life and mortality, (iii) a comorbidity for other life-threatening illnesses, (iv) a relatively simple diagnostic test, and (v) inexpensive treatment options [3]. Considering the stigma and difficulties in accessing quality healthcare faced by people with SCD living in developing countries, the disease falls into the category of NTD [4].

WHO—Africa declares SCD as a silent killer that requires urgent attention because in many countries, the newborn screening programs are very limited, difficult to implement, or non-existent. One hundred and twenty million people with SCD worldwide live in Africa, and around 1000 new babies are born every day with a high risk of SCD [5,6].

With an estimated prevalence of 3.5% for the homozygous form and 26.8% for the heterozygous, the Democratic Republic of Congo (DRC) is considered the second country in Africa and the third in the world, after India and Nigeria, respectively, most likely to develop new cases of SCD. Approximately 40,000 newborns are affected by the homozygous form each year [7,8,9,10].

Also called sickle cell anemia, SCD is a genetic disease that affects hemoglobin (1), with a specific mutation in the sixth codon of the *β*-globin gene that leads to the substitution of glutamic acid by valine, resulting in the polymerization of red blood cells (2) and causing anemia due to chronic hemolysis of the red blood cells (RBCs). Polymerization of RBCs causes the production of reactive oxygen species (ROS), activation of Toll-like receptor 4 (TLR4), and production of extracellular neutrophil traps (NETs), which lead to oxidative stress and related inflammation to the release of inflammasomes [3]. Apart from anemia, other clinical manifestations include vaso-occlusive crises, acute chest syndrome, kidney injury, stroke, splenic sequestration, and atherosclerosis [4].

Management of SCD principally involves the symptomatic treatment of patients to relieve pain, anemia, and vaso-occlusive crises, mainly using chemical substances, such as hydroxyurea, phytomedicines such as Niprisan^®^, or other herbal products that have proved efficacious in adults and children [11,12,13,14]. However, due to their high cost and their limited availability in the city of Kisangani, DRC, those products are not easily accessible to the sickle cell patients [15]. In some developed countries, bone marrow transplantation, a very effective technique, is offered but is not yet feasible in Kisangani due to its requirements and high cost [16].

This has led to the use of traditional medicinal plants for the management of SCD, including *Hibiscus tiliaceus* L., *Carica papaya* L., *Moringa oleifera* Lam., *Terminalia catapa* L., *Alchornea cordifolia* Müll.Arg., *Persea americana* Mill., *Morinda lucida* Benth., *Tectona grandis* L., *Theobroma cocoa* L., etc. Previous studies have described the evaluation of the antisickling activity of most of these plants, which justifies their current use [17,18,19]. However, in the case of *H. tiliaceus*, the most used in Kisangani by SCD patients, previous studies were focused on metabolic syndromes (diabetes and dyslipidemia) and the plant’s antidepressant, antimutagenic, and anticancer activities [20,21,22,23]. Additionally, there are some studies that describe the antibacterial, immunomodulatory, anti-inflammatory, antioxidant, and thrombolytic activities, which could partly justify its use in the case of sickle cell anemia [24,25,26,27,28,29]. The extracts were made with hexane, methanol, and ethanol, from which several metabolites were isolated, such as phenol acids, flavonoids, coumarins, and terpenoids [30,31,32,33].

**Figure 1:**
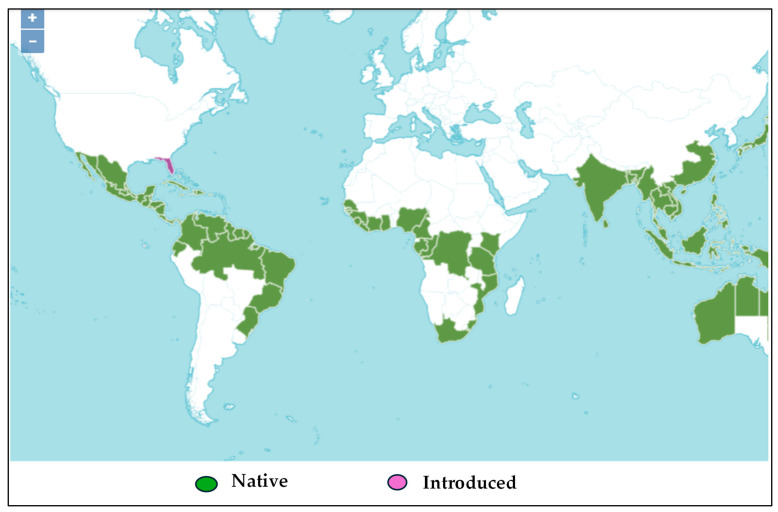
Geographical distribution of *H. tiliaceus* L. in the subtropical countries [34]. The green color indicates countries where *H. tiliaceus* originated, while the purple color indicates the countries where it has been introduced through cultivation.

*H. tiliaceus* is a Malvacaea plant widely distributed in several countries around the world (Figure 1). It can adapt to any soil type but has a preference for soils rich in organic matter and with high humidity. The *H. tiliaceus* leaves, which are mostly used to manage SCD by the local population, were collected from Kisangani, DRC. This study focused on a phytochemical analysis of the flavonoid and phenol acids in the aqueous extract of *H. tiliaceus* leaves and the evaluation of their biological activities against SCD. Our objective was to contribute to the improvement of SCD management in Kisangani and beyond by providing virtuous scientific evidence (biological activities) on the use of medicinal plants, such as *H. tiliaceus*.

## 2. Results and Discussion

### 2.1. Phytochemical Characterization

#### 2.1.1. Samples and Chemicals Compositions

**Figure 2:**
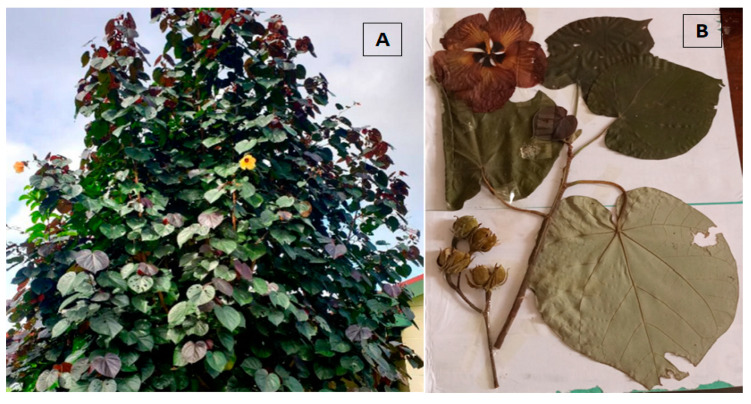
*H. tiliaceus* in its natural environment in Kisangani (**A**) and the herbarium specimen deposed at Meise Herbarium Garden (**B**).

After confirming the species identification at the Meise Botanic Garden in Belgium, with number BR0000026185842V (see Figure 2), seven separate batches of *H. tiliaceus* leaves, collected from different soil types (arid and marshy), were dried and powdered to obtain dried decoction extracts. The powders’ average moisture content was 8.5%, and the aqueous extraction of the different batches yielded an average of 15.7% dry extract (*n* = 7 extracts, standard deviation = 1.25%).

#### 2.1.2. Chromatographic Profiles of Flavonoids and Phenol Acids

**Figure 3:**
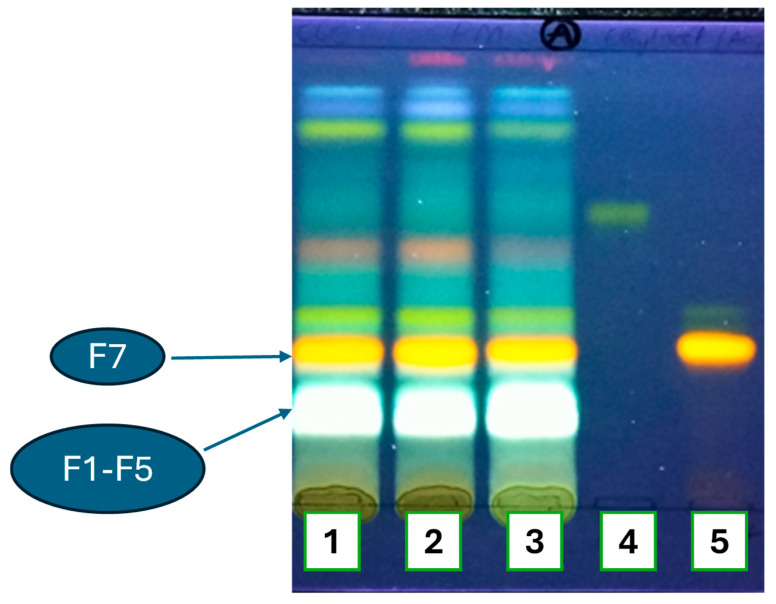
Thin-layer chromatographic profile of aqueous decoction extracts (1, 2, and 3) in presence of astragalin (4) and rutin (5). TLC conditions: silica gel plate; Mobile phase: ethyl acetate, glacial acetic acid, formic acid, water (100:11:11:26, *v*/*v*/*v*/*v*); Developer: Neu’s reagent; Detection: UV lamp at 366 nm.

A thin-layer chromatogram (TLC) analysis of the aqueous extracts (Figure 3) showed several spots under blue light and Neu’s reagent with various colors, among which yellow–orange and greenish white were observed, which are characteristics of flavonoids and caffeic acids derivatives, respectively. Further flavonoid identification allowed us to confirm the presence of rutin. However, other complementary TLC tests did not confirm caffeic acid nor chlorogenic or neo-chlorogenic acids). Due to the advantages of TLC (rapid and easy to implement in low-income countries and cities, such as Kisangani), we could consider this stage as our contribution to a first-line quality control of *H*. *tiliaceus*.

The green–white (named F1–F5) and yellow–orange (named F7) spots were more dense compared to the others. They were further analyzed with more efficient separative techniques, such as HPLC-UV/DAD and HPLC-MS.

**Figure 4:**
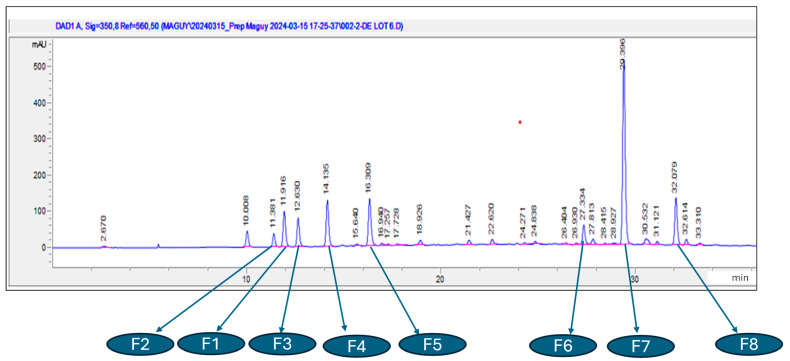
LC analysis of aqueous extract on Hypersil ODS C_18_ column (250 × 4.6 mm, 5 μm, 100 Å), using a gradient mode composed of acetonitrile (A) and ultra-pure water + trifluoroacetic acid 0.05% (B) as the mobile phase. Legend with the corresponding relative retention times of detected peaks. Unknown compounds of caffeic acid derivatives (at RRT = 0.340, F2 = 0.387, F1 = 0.405, F3 = 0.430, F4 = 0.481, and F5 = 0.555) and flavonoid (F6 = 0.930, F7 = 1.000, and F8 = 1.091). Only F7 was identified to be rutin.

HPLC-UV/DAD revealed several chromatographic peaks, among which were observed the derivative of caffeic acids (from 10 min to 20 min) and the flavonoids group (from 27 min to 33 min) (Figure 4). The high peak at about 29 min was confirmed to belong to rutin.

Preparative HPLC was performed to isolate the targeted compounds, mainly F1–F8, with the help of the TLC and HPLC-UV/DAD above used for monitoring and confirmation, respectively.

#### 2.1.3. Liquid Chromatography Coupled to Mass Spectrometry Analyses

**Figure 5:**
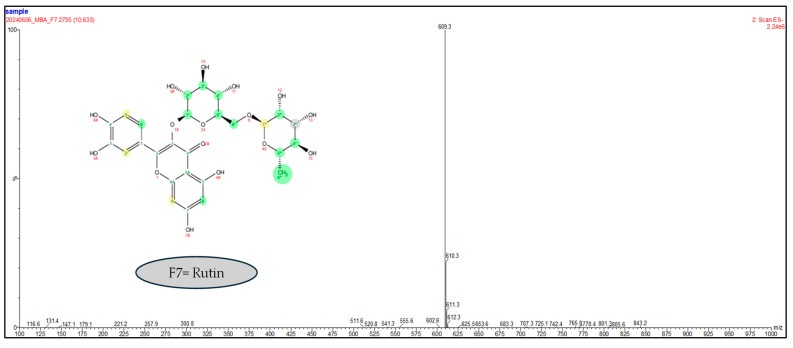
Negative mode LC-MS analysis of F7 with PFP column (150 mm × 4.6 mm, 100 Å, 5 µm), a flow rate of 0.3 mL/min, and an injection of 5 µL, in gradient mode, with the mobile phase composed of water + 0.1% formic acid and acetonitrile.

**Figure 6:**
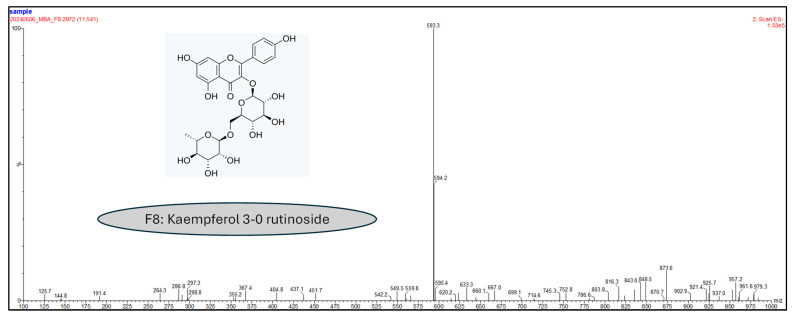
Negative mode LC-MS analysis of F8 with PFP column (150 mm × 4.6 mm, 100 Å, 5 µm), a flow rate of 0.3 mL/min, and an injection of 5 µL, in gradient mode, with the mobile phase composed of water + 0.1% formic acid and acetonitrile.

Since there was some doubt about the fractions’ purity, we resorted to LC-MS techniques for confirmation. Only fractions F1, F7, and F8 were isolated with 100% purity. Indeed, the masses obtained in the negative mode scan show the probable presence of rutin (609.3 g/moL) and kaempferol 3-*O*-rutinoside (593.3 g/moL) for fractions F7 (Figure 5) and F8 (Figure 6), respectively.

#### 2.1.4. Nuclear Magnetic Resonance Analyses

**Figure 7:**
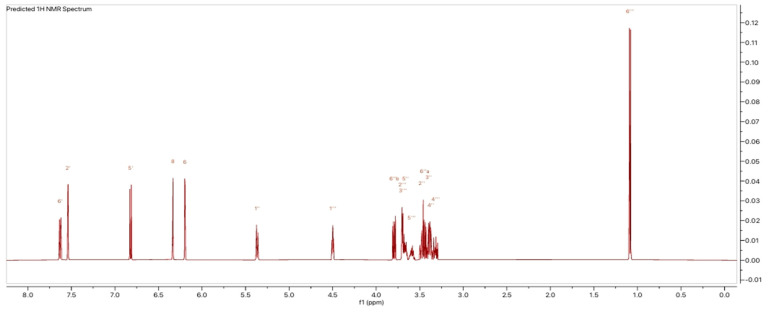
^1^H-NMR spectra of rutin F7 obtained from preparative HPLC of *H. tiliaceus* leaves; ^13^C NMR data for F7 were collected at 150 MHz in CD_3_OD; δ in ppm.

**Figure 8:**
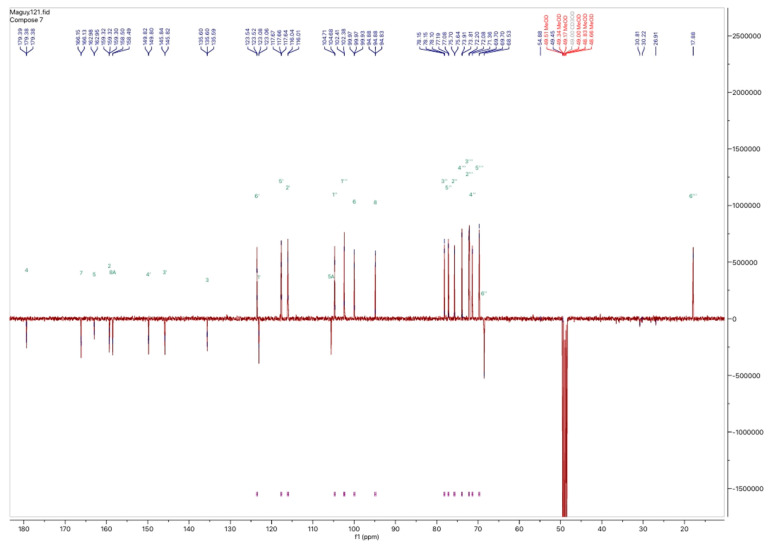
^13^C NMR spectra of rutin F7 obtained from preparative HPLC of *H. tiliaceus* leaves; ^13^C NMR data for F7 were collected at 150 MHz in CD_3_OD; δ in ppm.

In addition to the LC-MS analysis, fractions F7 and F8 were also examined using nuclear magnetic resonance (NMR) spectroscopy. Specifically, the proton and carbon-13 spectra were obtained for fraction F7. Figure 7 and Figure 8 illustrate the ^1^H and ^13^C NMR spectra of fraction F7, where the chemical shifts are indicative of rutin. The ^1^H NMR spectrum exhibited characteristic proton signals at 7.6, 6.8, 6.4, and 6.2 ppm, corresponding to five aromatic protons. The ^1^H NMR spectrum also endorsed glucose and rhamnose moieties at 5.4 ppm with the glucose anomeric proton signal and 4.5 ppm with the rhamnose anomeric proton signal. At 1.1 ppm (d, ^3^H, J = 6.6 Hz), a doublet corresponding to the methyl group of rhamnose was observed at a high field. The remaining protons resonated between 3.75 and 3.25 ppm in the sugar moiety. The ^13^C spectrum shows the different carbon positions on the genin part and on the sugar part, namely, glucose and rhamnose. We note the following: aglycone δ_C_ 159.3 (C-2), 135.6 (C-3), 179.3 (C-4), 162.9 (C-5), 99.9 (C-6), 166.1 (C-7), 94.8 (C-8), 158.5 (C-8A), 104.6 (C-5A), 123.0 (C-1′), 117.6 (C-2′), 145.8 (C-3′), 149.8 (C-4′), 117.6 (C-5′), 123,6 (C-6′); glucose moiety δ_C_ 104.6 (C-1″), 75.6 (C-2″), 77.0 (C-3″), 71.3(C-4″), 78.1(C-5″), 68.7 (C-6″); and rhamnose moiety δ_C_ 102.3 (C-1‴), 72.0 (C-2‴), 72.2 (C-3‴), 73.9 (C-4‴), 69.7 (C-5‴), 17.8 (C-6‴).

These features allowed the identification of the peak as rutin with a chemical structure of C_27_H_30_O_16_. This agrees with previous studies where rutin has been isolated and identified in plant extracts. Regarding the F8 fraction, in addition to the LC-MS data, we relied on data from previous studies that describe the identification of kaempferol 3-*O*-rutinoside in *H. tiliaceus* leaves’ extract. Other studies have identified rutin, isoquercitrin, trans-tiliroside, and kaempferol 3-*O*-glucoside (astragalin) in the leaves methanolic extracts [28,32].

This variation may result from several factors, including soil type, plant variety (distinguished by leaf or flower color), age, and harvest period. Exploring this aspect in future studies is crucial, as it impacts both the qualitative and quantitative profiles of the plant [35,36,37].

### 2.2. Biological Activities Related to the SCD Clinical Consequences

#### 2.2.1. Antisickling Test

This test showed that the F8 fraction had the best normalization capacity of red blood cells (RBC), up to 80% at a concentration of 50 µg/mL, unlike F7, which was limited to less than 30% normalization at all the tested concentrations. The test duration was up to 60 min.

**Figure 9:**
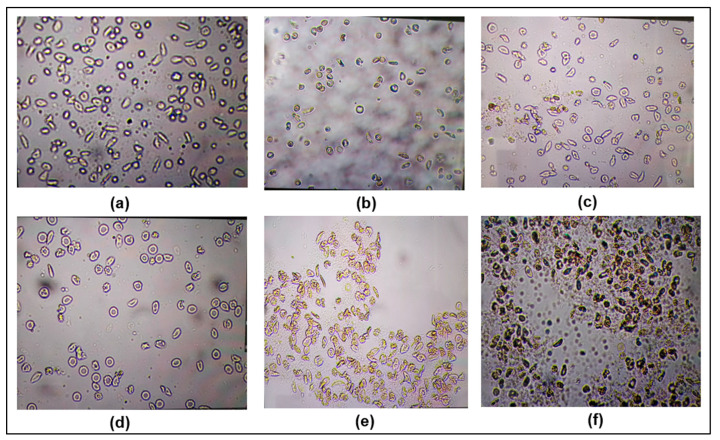
Morphology of erythrocytes observed using the 40× objective of a digital binocular microscope: untreated SS blood as the negative control (**a**), treated with 50 µg/mL of sample F1 (**b**), treated with 50 µg/mL of sample F7 (**c**), treated with 50 µg/mL of sample F8 (**d**), treated with 50 µg/mL of the total extract, (**e**) and treated with 100 µg/mL of the total extract (**f**).

However, at 100 µg/mL concentration, the images of the F8 sample and the total extract sample were difficult to interpret due to the undetectability of the RBCs. This situation suggests more likely RBC destruction when in contact with those samples. At that concentration, we also noted an unusual color change from red to gray greenish. This constitutes an important aspect to take care in the standardization of plant-based recipes in order to avoid adverse reactions of the pro-oxidant or hemolytic type. One can highlight this as virtuous scientific evidence to advise on the use of medicinal plants. Figure 9 presents the RBC appearance after sample processing under several testing conditions.

**Figure 10:**
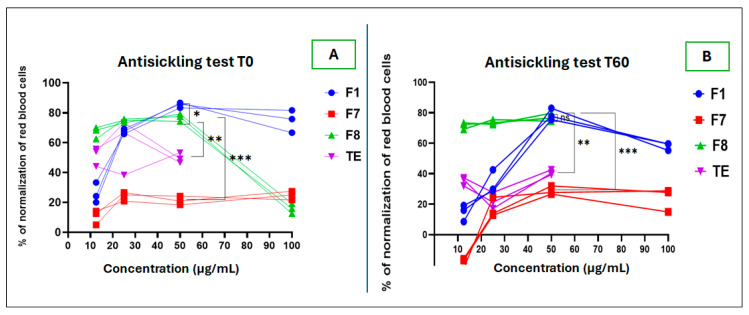
RBC normalization profiles of F1, F7, F8, and total extract (TE) at time 0 min (**A**) and 60 min (**B**) of test. Legend: (ns): *p* > 0.05; (*): *p* = 0.040; (**): *p* = 0.002; (***): *p* < 0.0002.

To compare the antisickling activity of different extract samples, it was necessary to determine the minimal concentration of RBC normalization. We also compared the RBC behavior for 60 min after treatment with the samples to be tested. Figure 10 shows that F8 presents the ability to normalize 50% of RBC at a concentration of <12.5 µg/mL. However, this normalization rate decreases over time to less than 40% for the F1 samples and the total extract, whereas for F8, the normalization rate was 70% at 12.5 µg/mL and remained almost unchanged after 60 min. Previous studies have shown that rutin at 100–500 pg/mL can prevent hemolysis, even in the presence of hemolytic substances, including phenylhydrazine used at 1–500 pg/mL. In addition, this antihemolytic effect highlights the pertinent and favorable activities of rutin as a promising molecule for improving the quality of life of sickle cell patients [38,39].

The antisickling activity of kaempferol extracted from *Justicia carnea*, methyl-kaempferol extracted from *Justicia secunda*, and kaempferol 3-*O*-glucoside extracted from *Uapaca heudelotii* Baill. has also been reported in previous studies. This reinforces the findings of our research and suggests that the ability of kaempferol heterosides to normalize sickle red blood cells is mediated through their aglycon moiety [40,41,42].

Conversely, it should be noted that this may vary for other activities. For instance, kaempferol 3-*O*-glucoside has demonstrated anti-inflammatory and antioxidant properties, whereas its rutinoside form does not. Similarly, rutin shows different activities depending on whether it is in its aglycon or glycosidic form [43,44,45].

#### 2.2.2. Antioxidant Tests

**Figure 11:**
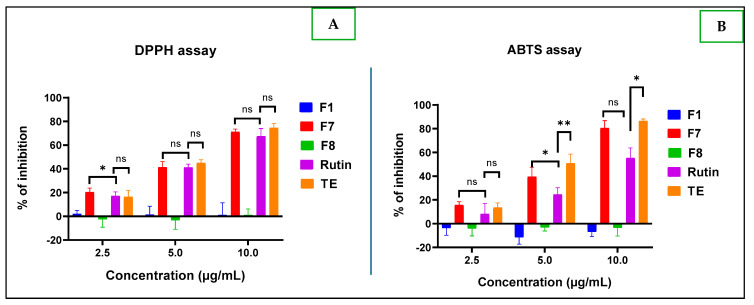
Antioxidant activities expressed as percentage of inhibition observed with DPPH test (**A**) and ABTS assay (**B**) of F1, F7, and F8, compared to rutin (used as a positive control) and the total extract (TE), as well. Legend: (ns): *p* > 0.05; (*): *p* < 0.05; (**): *p* = 0.004.

From Figure 11, it appears that only F7 has better concentration-dependent antioxidant power against DPPH and ABTS radicals compared to the reference compound rutin. Surprisingly, at the tested concentrations (from 2.5 to 10 µg/mL), F1 and F8 presented weak activity, even with reverse action, “pro-oxidant”, with the negative inhibition value exacerbated observed mainly with the ABTS test. This observation corroborates with previous research outcomes, where rutin showed antioxidant property with an IC_50_ of 17.57 ± 0.02 mM, and kaempferol 3-*O*-rutinoside had no activity [28]. One can highlight this as another virtuous scientific evidence to advise on the use of medicinal plants that contain such compounds. The antioxidant activity difference between the reference compound rutin and F7 fraction was statistically significant, with a *p*-value of 0.018 at a concentration of 5 µg/mL only for the ABTS assay.

#### 2.2.3. Anti-Inflammatory Test

**Figure 12:**
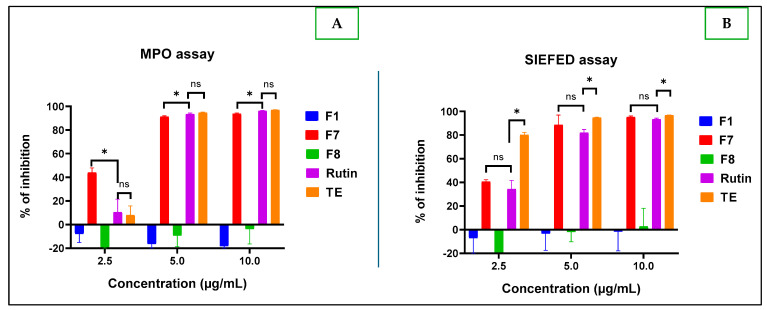
Anti-inflammatory profiles expressed as percentage of inhibition of MPO classic assay (**A**) and SIEFED assay (**B**) of the F1, F7, and F8 fractions compared to rutin (used as a positive control) and the total extract. Legend: (ns): *p* > 0.05; (*): *p* < 0.05.

It appears that at all the tested concentrations, the F1 and F8 fractions were slightly pro-inflammatory, unlike F7, which had better anti-inflammatory activity up to 80% at the minimum concentration of 5 µg/mL (Figure 12).

Figure 12A shows the ability of F7 to block the reaction catalyzed by myeloperoxidase (MPO). The difference between the anti-inflammatory activity of the F7 fraction and rutin was found to be statistically significant, with a *p*-value of 0.04 for the classical MPO test, and not statistically significant (*p* = 0.20) for the SIEFED test. In addition, Figure 12B shows the better myeloperoxidase inhibitory power of fraction F7 compared to rutin with no statistically significant difference. F7’s activity was also present even at 2.5 µg/mL, with 40% anti-inflammatory for both MPO direct assay (Figure 12A) and SIEFED (Figure 12B).

Indeed, myeloperoxidase (MPO) is an important enzyme that plays a crucial role in innate immunity stimulation. It regulates the production of reactive oxygen species, causing oxidative stress. Although this mechanism is essential for fighting infections, it also plays a key role in the pathogenesis of sickle cell disease [46]. Excessive MPO production increases oxidative stress, promoting inflammation and possibly contributing to the development of atherosclerosis [47,48,49,50]. Therefore, partial inhibition of MPO could improve the condition of sickle cell patients [51]. In previous research, kaempferol 3-*O*-rutinoside (F8) at 4 μM showed more effective antipyretic effects than ibuprofen and acetaminophen due to its ability to facilitate the elimination of inflammatory cytokines (IL-6 and TNF-α), whose levels can be used as a useful predictor for poor outcomes in SCD [52]. Rutin and kaempferol 3-*O*-rutinoside are two flavonols that differ by a single hydroxyl group, which is present in rutin but absent in kaempferol 3-*O*-rutinoside. Despite this difference, both remain heterosides containing a rhamnose and a glucose molecule. The absence of this hydroxyl group may reduce the anti-inflammatory and antioxidant properties of kaempferol 3-*O*-rutinoside compared to rutin. However, this structural variation enhances kaempferol’s ability to normalize sickle red blood cells through its aglycon moiety [53].

SCD and antioxidant/anti-inflammatory activities are connected by the crucial roles played by neutrophil–RBC interactions to promote vaso-occlusion, resulting in oxidative stress. Therefore, the inflammatory responses are the key components of numerous complications of the disease, including lysis of RBCs with the release of cell free hemoglobin (Hb) into the circulation and the subsequent production of reactive oxygen species. The immune cells, neutrophils, contribute to exacerbating inflammation through NETs and enzymatic reactions [54].

Polyphenols carry out their anti-inflammatory properties mainly through their antioxidant activity and their ability to inhibit enzymes involved in the production of eicosanoids. They decrease the activity of enzymes responsible for the generation of reactive oxygen species, such as xanthine oxidase and NADPH oxidase, while stimulating endogenous antioxidant enzymes, including superoxide dismutase, catalase, and glutathione peroxidase. Furthermore, they inhibit phospholipase A2, cyclooxygenase, and lipoxygenase, thereby limiting the production of prostaglandins and leukotrienes, which helps to attenuate inflammation. These mechanisms explain how aqueous extracts of *H. tiliaceus* provide lasting benefits to sickle cell patients, by spacing out crises, as described by the population of Kisangani [55].

## 3. Materials and Methods

### 3.1. Materials

#### 3.1.1. Vegetable Materials

*H. tiliaceus* L. belonging to the Malvaceae family was selected based on data from a survey conducted in 2022–2023 by Borive et al. [56] Leaves of *H. tiliaceus* were collected around the University of Kisangani campus in Kisangani, DRC. Plant identification was conducted at the herbarium of the Faculty of Science at University of Kisangani, with confirmation at the Botanical Garden of Meise in Belgium.

#### 3.1.2. Chemical Materials

The chemicals and the solvents: Glacial acetic acid, nitric acid, trifluoroacetic acid, formic acid, ethyl acetate, acetonitrile, ethanol, methanol, and deuterated methanol were of analytical grade from Merck VWR (Leuven, Belgium). Diphenyl borate aminoethanol (DPBAE); polyethylene glycol 400 (PEG); 2,2-Diphenyl-1-picrylhydrazyl (DPPH); 2,2 azino-bis(3-ethylbenzothiazoline)-6-sulfonic acid (ABTS); Amplex Red; sodium nitrite; sodium persulfate; hydrogen peroxide; rutin; and metabisulfite were all purchased from Sigma-Aldrich (Steinheim, Germany). Bovine serum albumin (BSA) was obtained from Roche Diagnostics Gmbh (Mannheim, Germany), and human myeloperoxidase (MPO) was purchased from Calbiochem Millipore (Bellirica, Madison, WI, USA).

### 3.2. Methods

#### 3.2.1. Preparation of Dry Aqueous Extracts

Due to quality assurance reasons for future experiments and traceability, it was important to ensure the identity of the raw material and its origin, as well. For these reasons, we collected fresh, young leaves of *H. tiliaceus* with the help of an experimented botanist of the Faculty of Sciences Herbarium (University of Kisangani).

The leaves of *H. tiliaceus* were immediately dried at room temperature in the laboratory of the Faculty of Medicine and Pharmacy at the University of Kisangani and ground to a powder using a ZM 200 ultra centrifugal electric grinder (Resch, France), then sieved through a 10 mesh sieve (2 mm of diameter). The resulting powders were packed in plastic vials, hermetically sealed, and sent to the University of Liège for splitting and characterization at the Pharmacognosy Laboratory (LPG) and biological testing at the Oxygen Center for Research and Development (CORD).

Aqueous extracts were prepared by adding 5 g of the powder to 100 mL of ultrapure water, then boiled at 100 °C on a hot plate for 15 min. The resulting solution was then filtered through glass wool and freeze-dried for 48 h.

#### 3.2.2. Separation by Column Chromatography

Qualitative HPLC analyses were performed on an HPLC-UV/DAD Agilent 6210 Infinity chromatograph equipped with a Hypersil column (250 mm × 4.6 mm i.d.), containing the ODS stationary phase (C_18_, 5 μm 100 Å), and an autosampler using a DAD detector set at 220 nm, 254 nm, 280 nm, and 350 nm. The flow was set to 1 mL/min, with an injection volume of 10 μL, using a gradient of acetonitrile (A) and ultrapure water + trifluoroacetic acid 0.05% (B): 0 min, 0:100 (A:B); 1 min, 3:97 (A:B); 45 min, 40:60 (A:B); 55 min, 40:60 (A:B); 56 min, 60:40 (A:B); 66 min, 60:40 (A:B); 67 min, 00:100 (A:B); 82 min, 00:100 (A:B) run–stop.

Preparative HPLC analyses were performed on a Varian chromatograph equipped with a Hypersil column (2.5 × 30 cm i.d.), containing the ODS stationary phase (C_18_, 12–15 μm 100 Å). The flow was set to 30 mL/min, with an injection volume of 10 mL.

#### 3.2.3. TLC Chromatography of Fractions

The fractions obtained were tested by TLC and HPLC-UV/DAD in order to evaluate their purity and select those to be analyzed by UPLC-QDa and NMR.

The HPLC-UV/DAD analysis was conducted as described above, while the TLC was performed on a G254 silica gel plate as the stationary phase and a mobile phase composed of a mixture of ethyl acetate, formic acid, acetic acid, and water (100:11:11:26 *v*/*v*/*v*/*v*). The resultant solution was created using Neu’s reagent.

#### 3.2.4. Analyses of Fractions Using LC-MS

The isolated fractions were analyzed by UPLC-QDa (Quadripole) from Waters (Antwerp, Belgium), and the compartments were Sample Manager FTN (SN M19FTP099G (Ver 1.71.395)), Quaternary Solvent Manager (SN A20QSP242A (Ver 1.72.415)) column compartment, QDa mass spectrometer (SN KBD5552 (Ver V), and PDA detector (SN C20UPD080A (Ver 1.70.63.67). The samples were prepared at 1 mg/mL in methanol of UPLC grade.The system was washed with 50:50 and 10:90 *v*/*v* mixtures of acetonitrile and water. Moreover, at the end of analyses, the column PFP (150 × 4.6 mm, 100 Å, 5 µm) was rinsed with a mixture of 65% methanol and 35% water for 1 h at a flow rate of 0.2 µL/min. The cone voltage was 30 V. The source and probe temperatures were set at 120 °C and 74 °C, respectively. The operating condition was as follows: run time of 32.5 min at a flow of 0.3 mL/min. The injection was 5 µL, and the mobile phase was composed of water + formic acid 0.1% and acetonitrile.

#### 3.2.5. Analyses of Fraction Using NMR

These analyses were performed in a Bruker Avance Neo 500 MHz spectrometer (Boston, MA, USA), equipped with a cryoprobe. The fractions were dissolved in deuterated methanol to achieve 30 mg/mL and put in NMR tubes (Cortec Net, Les Ulis, France). The ^13^C, DEPT-90, and DEPT-135 spectra were acquired with 6144 scan, and standard Bruker parameters were applied. The NMR spectra were displayed and interpreted using MestReNova software (version 15.0.1).

#### 3.2.6. Biological Activities Tests

##### Antisickling Activity

This test was assessed using the Emmel test, as described by Souleyman et al. [57], with slight modifications.

1. Treatment of sickle cell blood: Mix 500 µL of fresh blood with 500 µL of 2% sodium metabisulfite freshly prepared. Dilute the mixture obtained twice by adding 1000 µL of 0.9% NaCl.
2. Preparation of the samples to be tested: Dissolve 50 mg of extract in 5 mL of methanol 18%. From the latter, make several dilutions with 0.9% NaCl to obtain four different concentrations to test: 12.5, 25, 50, and 100 µg/mL.
3. By means of Eppendorf tubes, mix 100 µL of diluted treated blood with 100 µL of extract of the different concentrations.
4. Place 5 µL of the mixture obtained in step 3 on a slide, cover with a coverslip, and observe under a 40-objective microscope for the morphological analysis of the erythrocytes on four different fields (*n* = 2). Methanol 18% was used as a negative control and zinc oxide as a positive control.

The antisicking activity (AA) was expressed as follows:

AA = (P0 − P1)/P0 × 100 (1)

with P0 representing the mean of the control sickle cells and P1 representing the mean of the sickle cells in the samples tested.

##### Antioxidant Activity

- ABTS Assay

This test is based on the change of the blue–green color of the ABTS radical cation (ABTS^•+^) solution into its colorless neutral form, as previously described by Re et al. [58]. Briefly, the assay was performed by following two steps:

(a) Generation of ABTS^•+^ radicals: Separately prepare aqueous stock solutions of ABTS (7 mM) and sodium persulfate (2.45 mM), respectively. Mix and incubate in the dark for 12 to 16 h. Dilute the resulting solution in absolute methanol to adjust the absorbance to reach the value of 0.70 ± 0.02 at 734 nm, at 25 °C.
(b) Assay of antioxidant effect: Perform the analysis in triplicate using a microplate reader (Thermo Lab System, Vantaa, Finland). For each measurement, fill the well with 2 µL of the test compound solution and complete with 198 µL of ABTS^•+^ solution to reach a final volume of 200 µL; incubate for 30 min. Evaluate the absorbance of different solutions at 740 nm using a microplate reader (Thermo Lab system, Vantaa, Finland). For the controls, methanol and rutin were used as a negative control and a positive control, respectively. Determine the reducing capacity according to the following formula:

%inhibition = (A control − A sample) × 100/A control

with A representing absorbance.

- DPPH Assay

Following the method described by Brand Williams et al. [59], the DPPH assay was conducted in this way:

(a) Generation of DPPH^•^ radicals: Dissolve 3.2 mg of DPPH radical by stirring for 60 min in 100 mL absolute methanol to obtain a stock solution. Dilute the stock solution in methanol until an absorbance of 0.650 ± 0.020 at 517 nm is obtained. Perform the assay in triplicate on a multi-well plate.
(b) Assay of antioxidant effect: Fill each well with 2 µL of the tested compound and add 198 µL of the working DPPH^•^ solution. Incubate the mixture for 30 min in a dark place. In the presence of an antioxidant, the DPPH^•^radical is reduced to DPPH, resulting in a color change from purple to yellow. To quantify the effect, the decrease in absorbance at 510 nm was measured using a Multiskan Ascent 96 plate reader (Thermo Lab System, Vantaa, Finland). Methanol and rutin were used as a negative control and a positive control, respectively. Calculate the inhibition percentage of the DPPH^•^ radical using the same equation as the ABTS assay.

##### Anti-Inflammatory Activities

According to the method described by Nyssen et al. [60], the following steps were applied to perform both classical enzymatic and SIEFED (specific immuno-extraction followed by enzymatic detection) assays to assess anti-myeloperoxidase activity.

(a) Preparation of the enzyme solution: Dilute 4 µL of human myeloperoxidase in 8 mL of phosphate-buffered saline (20 mM PBS, pH 7.4), containing 5 g/L of BSA and 0.1% Tween-20.
(b) Preparation of the tested samples: Prepare the sample solution from the stock, dilute at final concentrations ranging from 2.5 to 10.0 µg/mL, and incubate for 10 min with the buffered solution of MPO (5 mU/mL).
(c) Preparation of resultant solution (AR/H_2_O_2_/NO_2_-): This solution is composed of Amplex Red (AR), hydrogen peroxide as a peroxidase substrate (10 µM), and sodium nitrite (4.5 mM), prepared separately in a phosphate buffer at pH 7.4, except H_2_O_2_, for which distilled water was used.
(d) MPO activity by classical assay: After incubation, measure the peroxidase activity by adding 10 µL of the sodium nitrite solution (4.5 mM, final concentration) and 100 µL of the reactional solution containing 10 µM hydrogen peroxide and 40 µM Amplex^®^ Red (AR) in phosphate buffer (50 mM) at pH 7.4 to each well (100 µL of the mixture) of the tested compound or vehicle (ultrapure water) mixed with MPO into a transparent multi-well plate. Monitor the oxidation of AR into the fluorescent resorufin adduct (excitation = 544 nm; emission = 590 nm) for 30 min at 37 °C with a fluorescent plate reader (Fluoroskan Ascent, Fisher Scientific, Hampton, NH, USA).
(e) MPO activity by SIEFED assay: Prepare samples with MPO and various concentrations of selected extract and incubate like in the classical assay. Load a mixture of 100 µL of each solution (MPO alone or MPO + extract) into the wells of a SIEFED microtiter plate (coated with rabbit polyclonal antibodies against human MPO and incubated for 2 h at 37 °C in darkness). Wash the wells four times with washing buffer, carefully dry the wells, and immediately measure the activity of the enzyme captured by the antibodies the same as for the classical assay, by adding 10 µL sodium nitrite solution (4.5 mM) and 100 µL of a reactional solution containing 10 µM hydrogen peroxide and 40 µM Amplex^®^ in phosphate buffer (50 mM) at pH 7.4. Monitor the oxidation of AR into the fluorescent resorufin adduct (excitation = 544 nm; emission = 590 nm) for 30 min at 37 °C with a fluorescent plate reader (Fluoroskan Ascent, Fisher Scientific, Hampton, NH, USA). As for the MPO direct assay, a control assay set as the relative value of MPO activity was performed with purified MPO in the presence of PBS instead of the samples. For both MPO assays, calculate the inhibition percentage using the following formula:

%inhibition = (Acontrol − Asample) × 100/Acontrol

with A representing absorbance.

#### 3.2.7. Statistical Analysis

All the data were subjected to statistical analysis using GraphPad Prism 8.0.1 software, developed by the company GraphPad. The values were reported as mean ± standard deviation. Two-way ANOVA multiples comparisons test and Dunnett’s post-tests were used to test the differences between the treatment groups. The results were considered significant at *p*-values of less than 0.05, that is, at 95% confidence level.

## 4. Conclusions

This study focused on SCD, classified by WHO—Africa as an NTD. Our goal was to provide scientific evidence for the use of *H. tiliaceus* to fight against the SCD clinical symptoms. Several analytical profiles, including the TLC profile of the aqueous extract, which enabled the identification of rutin, were established. The LC-UV/DAD and LC-MS analyses confirmed the presence of rutin and kaempferol 3-*O*-rutinoside. Rutin was further validated using NMR. These analytical techniques were deemed valuable for different purposes: TLC for preliminary field characterization, HPLC-UV/DAD for laboratory-level confirmation, and LC-MS and NMR for advanced characterization.

*H. tiliaceus* aqueous extracts presented interesting and promising antisickling biological activity with interesting RBC normalization. The interesting antioxidant effect observed was due to the presence of rutin, while kaempferol 3-*O*-rutinoside had a pro-oxidant effect at the concentration tested. The anti-inflammatory test was very satisfactory since the percentage inhibitions with MPO and SIEFED were achieved at 2.5 µg/mL.

Our study provides relevant information on the ability of *H. tiliaceus* aqueous extracts against SCD symptoms. To the best of our knowledge, it is the first time kaempferol 3-*O*-rutinoside’s antisickling effect has been confirmed.

## Data Availability

Data are contained within the article.

## References

[1] World Health Organization. https://www.who.int/news-room/questions-and-answers/item/neglected-tropical-diseases.

[2] Hotez P.J., Aksoy S., Brindley P.J., Kamhawi S. (2020). What constitutes a neglected tropical disease?. PLoS Negl. Trop. Dis..

[3] Ware R.E. (2013). Is Sickle Cell Anemia a Neglected Tropical Disease?. PLoS Negl. Trop. Dis..

[4] Tshilolo L., Gonzalez J.P. (2024). Stigmatization of sickle cell disease across the Democratic Republic of Congo: A presentation of two cases. Int. Health Trends Perspect..

[5] World Health Organization. https://www.afro.who.int/health-topics/sickle-cell-disease.

[6] Williams T.N. (2016). Sickle Cell Disease in Sub-Saharan Africa. Hematol. Oncol. Clin. N. Am..

[7] Tshilolo L., Aissi L.M., Lukusa D., Kinsiama C., Wembonyama S., Gulbis B., Vertongen F. (2009). Neonatal screening for sickle cell anaemia in the Democratic Republic of the Congo: Experience from a pioneer project on 31 204 newborns. J. Clin. Pathol..

[8] Katawandja A.L., Kingwengwe A.A., Shindano E.M. (2020). Knowledge and Practices of Health Providers on the Diagnosis and Biological Monitoring of Sickle Cell Disease in the City of Kindu, in the East of the Democratic Republic of Congo. OALibrary.

[9] Kasai E.T., Boemer F., Marini R.D., Kadima J.N., Batina S.A., Ngbonda N.D., Alworong’a J.P.O. (2020). Systematic Screening of Neonatal Sickle Cell Disease with HemoType Sickle cell Kit-Test: Case Study and Literature Review. Open J. Blood Dis..

[10] Shongo Y.P.M. (2022). Sickle cell disease in the Democratic Republic of Congo/DRC: Burden of a heavier price. J. Med. Public Health.

[11] Akinyemi O.D.O., Oguche S., Kehinde A., Edache S.O., Tolulope O.A., Ijeoma N.D., Ayuba I.Z., Atiene S.S. (2020). Effectiveness and Safety of Hydroxyurea in the Treatment of Sickle Cell Anaemia Children in Jos, North Central Nigeria. J. Trop. Pediatr..

[12] Wambebe C.O., Bamgboye E.A., Bidemi O.B., Hadiza K., Jafaru A.M., Ekpeyong M., Benedict S.A., Njoku S.O., Nasipuri N.R., Olubayo O.K. (2001). Efficacy of Niprisan in the prophylactic management of patients with sickle cell disease. Curr. Ther. Res..

[13] Cordeiro N.J.V., Oniyangi O. (2004). Phytomedicines (medicines derived from plants) for sickle cell disease. Cochrane Database Syst. Rev..

[14] Tshilolo L., Tomlinson G., Williams T.N., Brígida S., Olupot-Olupot P., Lane A., Banu A., Susan E.S., Latham T.S., Ware R.E. (2019). Hydroxyurea for Children with Sickle Cell Anemia in Sub-Saharan Africa. N. Engl. J. Med..

[15] Mukinayi B.M., Cibeyibeyi G.K., Disashi G.T., Gulbis B. (2021). Sickle cell disease in the Democratic Republic of Congo: What are the obstacles to treatment with hydroxyurea?. Pan. Afr. Med. J..

[16] Bernaudin F. (2019). Why, Who, When, and How? Rationale for Considering Allogeneic Stem Cell Transplantation in Children with Sickle Cell Disease. J. Clin. Med..

[17] Nurain I.O., Bewaji C.O., Jarrett S.J., Robertson D.D., Zhang Y. (2017). Potential of Three Ethnomedicinal Plants as Antisickling Agents. Mol. Pharm..

[18] Ibrahim S., Ukwuani-Kwaja A.N., Maimuna H. (2021). Ethnobotanical Survey and In vitro Antisickling Effect of Some Selected Medicinal Plants. Asian J. Res. Biochem..

[19] Yembeau N.L., Biapa Nya P.N., Pieme C.A., Tchouane K.D., Kengne Fotsing C.B., Nya Nkwikeu K.J., Feudjio A.F., Telefo P.B. (2022). Ethnopharmacological Study of the Medicinal Plants Used in the Treatment of Sickle Cell Anemia in the West Region of Cameroon. Evid.-Based Complement. Altern. Med..

[20] Jeffery T.D., Matthew L.R. (2021). A review of the effectiveness of hibiscus for treatment of metabolic syndrome. J. Ethnopharmacol..

[21] Abdul-Azeez Z.M., Shihab H.M. (2023). Possible Protective Anticancer effect of Ethanol Fraction of Iraqi *Hibiscus tiliaceus* L. Leaves Extract on Diethylnitrosamine-induced Hepatocarcinogenesis in Male Rats. Iraqi J. Pharm. Sci..

[22] Rosa R.M., Melecchi M.I., Halmenschlager R.C., Abad F.C., Simoni C.R., Carama E.B., Gashenriques J.T., Affi J.S., Ramos A. (2006). Antioxidant and Antimutagenic Properties of *Hibiscus tiliaceus* L.. Methanolic Extract. J. Agric Food Chem..

[23] Vanzella C., Bianchetti P., Sbaraini S., Vanzin S.I., Melecchi M.I.S., Caramão E.B., Siqueira I.R. (2012). Antidepressant-like effects of methanol extract of *Hibiscus tiliaceus* flowers in mice. BMC Complement. Altern. Med..

[24] Andriani Y., Hanifah W., Kholieqoh A.H., Majid F.A.A., Hermansyah H., Amir H., Muhammad T.S.T. (2023). Antibacterial activity of hexane and methanol fractions of some selected plants against *Klebsiella pneumoniae*. J. Adv. Pharm. Technol. Res..

[25] Trung V.T., Linh K.T.P., Thu Trang D., Thanh Binh P., The Cuong N., Thanh N.V., Thao N.P. (2023). Antimicrobial constituents from the leaves of *Hibiscus tiliaceus* L.. Nat. Prod. Res..

[26] Surana A.R., Kumbhare M.R., Gunjal A.R., Goswami S.S., Ghuge D.M. (2022). Chemical characterization, thrombolytic and antioxidant activity of *Hibiscus tiliaceus* L. leaves. Nat. Prod. Res..

[27] Abdul-Awal S.M., Nazmir S., Nasrin S., Nurunnabi S.R., Uddin S.J. (2016). Evaluation of pharmacological activity of *Hibiscus tiliaceus*. Springer Plus.

[28] Vinh L.B., Nguyet N.T.M., Thanh C.D., Huong T.T., Tram L.H., Van Thong N., Kim Y.H. (2021). Chemical constituents of Vietnamese mangrove *Hibiscus tiliaceus* with antioxidant and alpha-glucosidase inhibitory activity. Nat. Prod. Res..

[29] Hidayati D.N., Maghfiroh H.R., Safitri A. (2023). Antibacterial activity of ethanol extracts of *Hibiscus tiliaceus* L. leaves from different extraction methods against Escherichia coli and Staphylococcus aureus. Pharmaciana.

[30] Chen J.J., Huang S.Y., Duh C.Y., Chen I.S., Wang T.C., Fang H.Y. (2006). A New Cytotoxic Amide from the Stem Wood of *Hibiscus tiliaceus*. Planta Med..

[31] Bassant M.M.I., Marawan A.E., Doha H.A.B., Emad N.Z., Souad E.G., Mouchira A.S. (2024). A pharmacological and toxicological biochemical study of cardiovascular regulatory effects of hibiscus, corn silk, marjoram, and chamomile. Heliyon.

[32] Lê Huyền T., Trần T.H., Nguyễn V.T., Nguyễn H.M. (2021). Flavonoid Glycoside Constituents from the Leaves of *Hibiscus tiliaceus*. JST Eng. Technol. Sustain. Dev..

[33] Bamba Fall A., Vanhaelen-Fastré R., Vanhaelen M., Lo I., Toppet M., Ferster A., Fondu P. (1999). In Vitro Antisickling Activity of a Rearranged Limonoid Isolated from *Khaya senegalensis*. Planta Med..

[34] Govaerts R., Nic Lughadha E., Black N. (2021). The World Checklist of Vascular Plants, a continuously updated resource for exploring global plant diversity. Sci. Data.

[35] Chaouqi S., Moratalla-López N., Alonso G.L., Lorenzo C., Zouahri A., Asserar N., Haidar E.M., Guedira T. (2023). Effect of Soil Composition on Secondary Metabolites of Moroccan Saffron (*Crocus sativus* L.). Plants.

[36] Mudau H.S., Mokoboki H.K., Ravhuhali K.E., Mkhize Z. (2022). Effect of Soil Type: Qualitative and Quantitative Analysis of Phytochemicals in Some Browse Species Leaves Found in Savannah Biome of South Africa. Molecules.

[37] Pant P., Pandey S., Dall’Acqua S. (2021). The Influence of Environmental Conditions on Secondary Metabolites in Medicinal Plants: A Literature Review. Chem. Biodivers..

[38] Ramani S., Basak S., Puneeth S., Karthick M., Malathi R. (2021). Anti Hemolytic Activity of Rutin in case of Phenyl Hydrazine induced Hemolysis. Curr. Trends Biotechnol. Pharm..

[39] Muhammad A., Waziri A.D., Forcados G.E., Sanusi B., Sani H., Malami I., Abubakar I.B., Oluwatoyin H.Y., Adinoyi O.A., Mohammed H.A. (2019). Sickling-preventive effects of rutin is associated with modulation of deoxygenated haemoglobin, 2,3-bisphosphoglycerate mutase, redox status and alteration of functional chemistry in sickle erythrocytes. Heliyon.

[40] Gbolo B.Z., Ngbolua K.N., Ciala B.N., Semay I., Mpiana P.T., Gerbaux P., Duez P. (2024). LC-MS/MS Analysis of crude Flavonoid Compounds from *Justicia secunda* from Democratic Republic of the Congo and evaluation of their antisickling Activity. Nat. Resour. Hum. Health.

[41] Ekutsu G.E., Kitete E.M., Masengo C.A., Gbolo B.Z., Iteku J.B., Mavakala B.K., Mpiana P.T., Mudogo V., Ngbolua K.N. (2024). Molecular Docking Simulation of the antisickling activity of Naringenin-7-*O*-glucoside and Kaempferol-3-O-glucoside from *Uapaca heudelotii* Baill. (Phyllanthaceae) and their ADMET profile. J. Oral Public Health.

[42] Igbinumwen O., Osaro I., Ebengho M.I., Edema M.O., Oviawe A.P., Momoh S.M., Eduwuirofo L.O., Iyekekpolor R., Oghomwen T.M., Oghomwen R.O. (2024). Chemical characterizations and anti-sickling potential of methanol extract of *Justicia carnea* (flamingo plant). Zenodo.

[43] Hu X., Wang M., Pan Y., Xie Y., Han J., Zhang X., Niayale N., He H., Li Q., Zhao T. (2020). Anti-inflammatory Effect of Astragalin and Chlorogenic Acid on *Escherichia coli*-Induced Inflammation of Sheep Endometrial Epithelium Cells. Front. Vet. Sci..

[44] Park H.M., Lee J.Y., Kim M.Y., Kang C.-H., Hwang H.S. (2021). Anti-Oxidative and Anti-Inflammatory Activities of *Astragalus membranaceus* Fermented by *Lactiplantibacillus plantarum* on LPS-Induced RAW 264.7 Cells. Fermentation.

[45] Choi S.-S., Park H.-R., Lee K.-A. (2021). A Comparative Study of Rutin and Rutin Glycoside: Antioxidant Activity, Anti-Inflammatory Effect, Effect on Platelet Aggregation and Blood Coagulation. Antioxidants.

[46] Poret M., Tran T., Villotte M., Nüsse O. (2017). Myeloperoxidase: An ingenious defense mechanism against pathogen infections in medicine and science. Med. Sci..

[47] Nur E., Biemond B.J., Otten H.M., Brandjes D.P., Schnog J.J.B. (2011). Oxidative stress in sickle cell disease; pathophysiology and potential implications for disease management. Am. J. Hematol..

[48] Queiroz R.F., Lima E.S. (2013). Oxidative stress in sickle cell disease. Rev. Bras. Hematol. E Hemoter..

[49] Cavalcante J.E., Machado R.P., Laurentino M.R., Dos Santos T.E., Bandeira I.C., Maia Filho P.A., Figueiredo M.F., Martins A.M., Lemes R.P. (2016). Clinical events and their relation to the tumor necrosis factor-alpha and interleukin-10 genotypes in Sickle-Cell-Anemia patients. Hematol. Oncol. Stem Cell Ther..

[50] Domingos I.F., Pereira-Martins D.A., Sobreira M.J.V.C. (2020). High levels of proinflammatory cytokines IL-6 and IL-8 are associated with a poor clinical outcome in sickle cell anemia. Ann. Hematol..

[51] Zhang H., Xu H., Weihrauch D., Jones W.D., Jing X., Shi Y., Gourlay D. (2013). Inhibition of myeloperoxidase decreases vascular oxidative stress and increases vasodilatation in sickle cell disease mice. J. Lipid Res..

[52] Zheng W., Wang H., Wang X., Li X., Hu J., Zi X., Zhou Y., Pan D., Fu Y. (2024). Kaempferol 3-O-Rutinoside, a Flavone Derived from *Tetrastigma hemsleyanum* Diels et Gilg, Reduces Body Temperature through Accelerating the Elimination of IL-6 and TNF-α in a Mouse Fever Model. Molecules.

[53] Wadah O., Mona S.M., Hassan S.K., Abdelkhalig M., Shantier S.W., Bashier O., Abdoon I. (2020). HPTLC Fingerprint Profile and Identification of Antidiabetic and Antioxidant Leads from *Bauhinia rufescens* L.. Adv. Pharmacol. Pharm. Sci..

[54] Hounkpe B.W., Chenou F., Domingos I.F., Cardoso E.C., Costa Sobreira M.J.V., Araujo A.S., Lucena-Araújo A.R., da Silva Neto P.V., Malheiro A., Fraiji N.A. (2021). Neutrophil extracellular trap regulators in sickle cell disease: Modulation of gene expression of PADI4, neutrophil elastase, and myeloperoxidase during vaso-occlusive crisis. Res. Pract. Thromb. Haemost..

[55] Yahfoufi N., Alsadi N., Jambi M., Matar C. (2018). The Immunomodulatory and Anti-Inflammatory Role of Polyphenols. Nutrients.

[56] Borive A.M., Mavar M.H., Memvanga B.P., Ndezu A.R., Nsasi B.E., Mouithys M.A., Batina A.S., Marini D.R. (2025). Evidence-based Sickle Cell management: From Traditional remedies use to laboratory assessment. Phytomed. Plus.

[57] Souleymane H.D., Djibo A.K., Seyni S.H., Zakaria O., Botezatu A.V., Dinica R.M., Ibrahim Maman Laouali A., Kouakou N.D.V. (2023). Phytochemical Characterization and In Vitro Evaluation of the Anti-Sickle Cell Activity of Aqueous and Ethanolic Extracts of Two Medicinal Plants from Niger: *Flueggea virosa* (Roxb. ex Willd.) Royle and *Kigelia africana* (Lam.) Benth. Plants.

[58] Re R., Pellegrini N., Proteggente A., Pannala A., Yang M., Rice-evans C. (1999). Antioxidant activity applying an improved ABTS radical cation decolorization assay. Free Radic. Biol. Med..

[59] Brand-Williams W., Cuvelier M.E., Berset C. (1995). Use of a free radical method to evaluate antioxidant activity. LWT Food Sci. Technol..

[60] Nyssen P., Mouithys-Mickalad A., Minguet G., Sauvage E., Wouters J., Franck T., Hoebeke M. (2018). Morphine, a potential inhibitor of myeloperoxidase activity. Biochim. Et Biophys. Acta (BBA) Gen. Subj..

